# Circadian Rhythm, Lifestyle and Health: A Narrative Review

**Published:** 2018-08

**Authors:** Dariush FARHUD, Zahra ARYAN

**Affiliations:** 1. School of Public Health, Tehran University of Medical Sciences, Tehran, Iran; 2. Dept. of Basic Sciences/Ethics, Iranian Academy of Medical Sciences, Tehran, Iran; 3. Genetics Clinic, Valli-e-Asr Sq., Tehran, Iran

**Keywords:** Lifestyle, Circadian rhythm, Health, Cosmos spin

## Abstract

**Background:**

The circadian rhythm regulation plays a crucial role in people’s healthy lives affected by factors consisting of cosmic events related to the universe and earth, environmental factors (light, night and day duration, seasons) and lifestyles. These factors changes lead to disturbance of circadian rhythm and it causes increasing the incidence of mental diseases like depression and physiological problems like cancers, cardiovascular disease and diabetes.

**Methods:**

The present review searched Elsevier, SID, Pub Med, Springer, and Google Scholar databases for relevant articles were published between 1996 and 2017. Keywords used included lifestyle, circadian rhythm, cancer, metabolic diseases and cosmic factors.

**Results:**

Circadian rhythm can be affected by lifestyle, heredity, cosmos spin and seasonal factors. Two first factors have physically direct effects on circadian rhythm and health, while other factors influence on them mentally. After all, all of them lead to cancer, cardiovascular diseases and metabolic obesity.

**Conclusion:**

Although environmental factors are universal events which are unrelated to human control but affect human’s body and circadian rhythm. Other factors are manageable by human to prevent disturbance of circadian rhythm making physical disorders.

## Introduction

Operation of the circadian clock, which is an internal regulator in cells of organisms, coordinates physiological and behavioral activities with daily environmental variations within 24 h cycles. In humans, dysfunction or misalignment of the circadian clock with environmental cues alters the timing of the sleep-wake cycle, leading to a variety of circadian rhythm sleep disorders (1).

Jeffrey Hall and Michael Rosbash of Brandeis University and Michael Young of Rockefeller University received the Noble Prize in 2017 in Physiology and Medicine, for extending work on how time is measured each day in biological system which also includes one’s own bodies. Now it has been appreciated that the central role of circadian rhythms plays in coordinating organism’s life with earth’s day, controlling everything from the metabolism to the sleep time. Understanding the powerful regulation of biology by circadian rhythms is beginning to lead to changing life’s management.

Adjusting our plans to the biological rhythms might beneficially influence our activities at work and school instead of programing our sleep schedules into routines which require waking up in the early morning. Moreover, one of the processes regulated by the circadian clock is the cell cycle. Therefore, cancerous cells which undergo abnormal cell division are due to disruption of circadian rhythms. A relationship was concluded between circadian rhythms changes and tumor-igenesis in some cancers and adenocarcinoma (2). Circadian rhythm has been associated with cardiovascular diseases (CVD) and its risk factors, including diabetes and obesity, on multiple levels, as well.

**Fig. 1:**
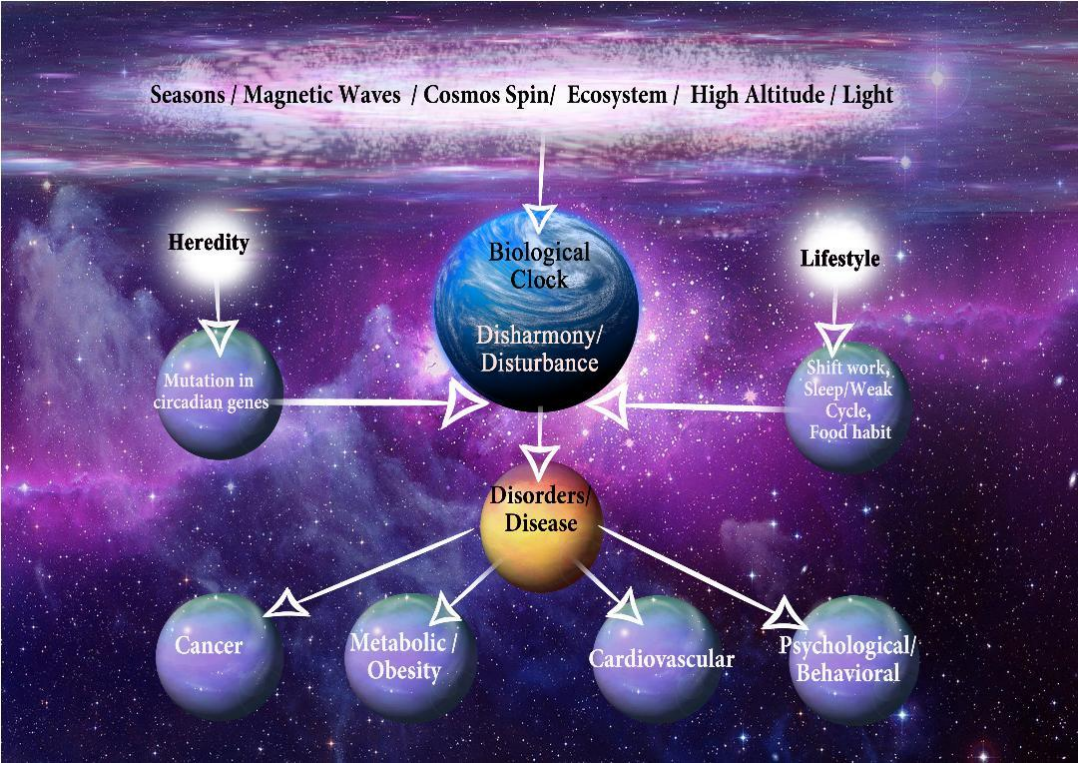
Basic mechanism of circadian rhythm disruption; several environmental and hereditary factors can cause disruption of the circadian rhythms (Original)

In this paper, we discuss the relationship among circadian rhythm, lifestyle and noncomunicable diseases. Fig. 1 shows our ideas in details.

## Methods

We searched Elsevier, SID, Pub Med, Springer, and Google Scholar databases for articles published between 1996 and 2017. We used various keywords such as “biological clocks, lifestyle, circadian rhythm, cancer, metabolic diseases and cosmos events”. Overall, 39 articles were included in the study. Investigation of original reasons for increasing cancer, metabolic obesity and cardiovascular diseases to find much better ways for curing them is the main idea of this review. We tried to search articles which discussed any factors and their results. Then, we gathered information and analyzed them to show relationships between those factors and their complications.

## Results and discussion

### Molecular Process of Circadian Rhythm

From Darwin’s finches on the Galápagos Islands to modern city dwellers, organisms adapt to their environment (Survival of the Fittest). Existence of 24 h day and night cycles on earth, caused evolution of circadian rhythms in the body’s cells. These phenomena help us determine when to rest, eat, or anticipate danger or predation.

**Fig. 2:**
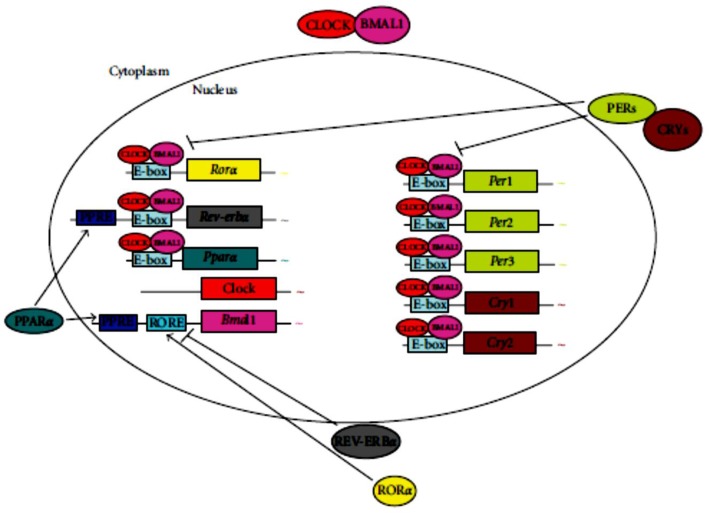
The core mechanism of the mammalian circadian clock and its link to energy metabolism. The cellular oscillator is composed of a positive limb (CLOCK and BMAL1) and a negative limb (CRYs and PERs). CLOCK and BMAL1 dimerize in the cytoplasm and translocate to the nucleus (5)

The start of modern circadian biology field goes back to the 1970s, when geneticist Seymour Benzer and his student Ron Konopka engaged in studying of the genes that encode biological timing in *Drosophila*, fruit flies. Several homologues of the core clock proteins in *Drosophila*, including CLK and PER, play similar roles in mammalian circadian timekeeping (3). With that gene in their sights, the labs of Hall, Rosbash and Young made the molecular era of circadian biology as the dissolved molecular mechanisms of biological time-keeping. The gene named PER encodes a protein which was discovered by Jeffry Hall and Michael Rosbash. It increases during the night and decreases during the day. The levels of this protein may play a crucial role to inform the cell what time it is. Such a negative feedback system is similar to how a thermostat controls the temperature of a place. If the temperature decreases below the set point, the thermostat turns on the heater. On the contrary, when the place gets hot, the thermostat turns off the furnace. Here, what works to control the heater and keep a constant temperature is the negative feedback. Hall and Rosbash reason this PER protein might actually block the activity of the period gene through turning itself off each day. Levels of PER gradually increase over the course of the night. Ultimately the protein levels fall and the process starts over again which is called a negative feedback loop. It is the same type of biological balance act that keeps everything from blood sugar levels to circadian rhythms in line throughout the body (4)(Fig. 2).

### The Interaction between Lifestyle and Circa-dian Rhythm

“Lifestyle is a set of goals, plans, values, attitudes, behaviors, and beliefs manifested in the personal and family life of the individual and in his social and cultural interactions. It is an interdisciplinary concept which involves a health-oriented view of the physical, psychological, social, and spiritual aspects of life” (6). Lifestyle has an interaction with the social and cultural structure and context. In this case, lifestyle consists of daily common activities of an individual such as sleep and waking time.

It will influence on people’s healthy lifestyle and even, the national economic gain. Studies showed that a simply delaying school start to 8.30 a.m. (one additional hour of sleep) could lead to an economic gain of $83 billion to the US economy within a decade, in terms of benefiting the public health of adolescents and doing so in a cost-effective manner. The earnings planed through the study’s economic model would be realized through the higher academic and professional performance of students, and reduced car crash rates (6). The most important disorders of circadian rhythm are sleep disturbance and depression which changing work time causes them. Physiological activities of body, like heart beat and excretion of epinephrine and norepinephrine hormones, adapt with cycle (7). But it is likely to last for others during a few days or weeks. People are different in accordance with work shift. The majority of people would be hurt partly or pass through the problems. On the contrary, others are feeling it hard to adapt to this disturbance in circadian rhythm and to their inner body’s circadian rhythm in comparison with their outer environment. It causes problems while they are doing daily activities.

**Fig. 3:**
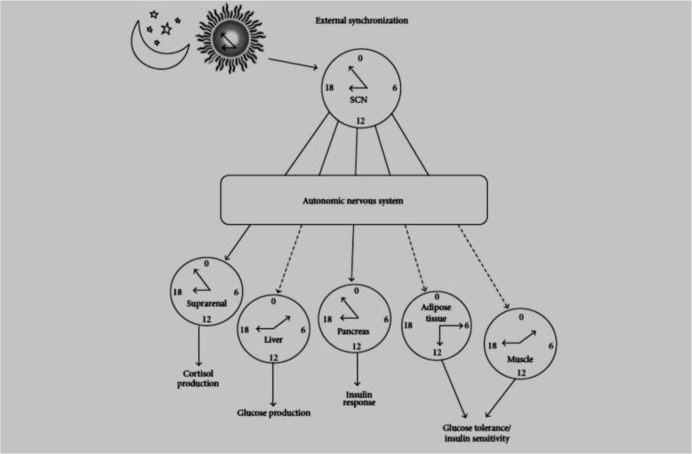
Schematic representation of a state of internal desynchronization. The biological clock (SCN) is synchronized with the dark-light cycle (external synchronization), and, in turn, the SCN (Suprachiasmatic Nucleus) synchronizes peripheral oscillators (internal synchronization) (16)

Today, modern technology developing makes social and work activities to be independent of the environment’s light/dark duration. Hence, long flights across the continents (jet-lag), shift work and night work as kinds of habits of modern lives can influence circadian rhythm function (8–10). The exposure to artificial light, for short periods of time during the night causes a significant shift in the circadian rhythms, which leads to irritability, anxiety and depressive behaviors, also decreasing learning and memory efficiency in animal models (11–15). In the hypothalamus there is a region called Suprachiasmatic Nucleus (SCN) above the optic chiasma. Studies have indicated that SCN controls circadian rhythms by receiving signals from photic signals which influence the biological clock (16) (Fig. 3).

### The Hormonal Mechanism of Circadian Rhythm

**Fig. 4:**
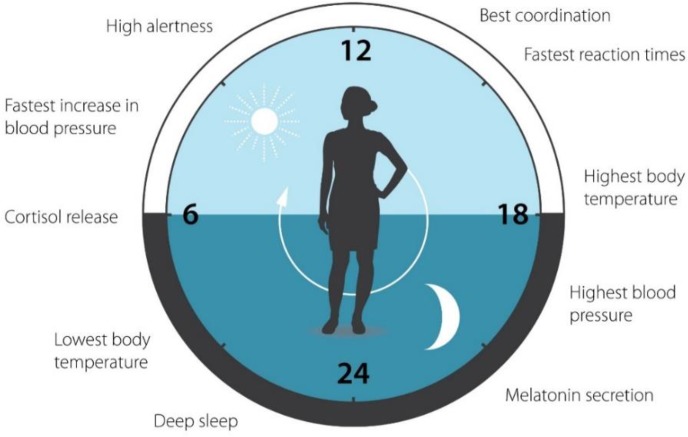
The circadian clock has an impact on many aspects of our physiology. This clock helps to regulate sleep patterns, feeding behavior, hormone release, blood pressure and body temperature. A large proportion of our genes are regulated by the clock (18)

Melatonin, a hormone secreted by the Pineal Gland, is one of the major signaling molecules used by the master circadian oscillator to entrain downstream circadian rhythms. Its secretion is affected by factors such as age, light, environmental and physiological factors (17) (Fig. 4). Also, genetic disorders of the melatonin receptor is related to biochemical disturbance in glucose metabolism pathway which ends to increase the risk of developing type 2 diabetes (19). Moreover, declining melatonin secretion speeds up aging and tumorogenesis, visceral adiposity and cardiovascular function (20–22).

### The Relationship between Lifestyle and Diseases (Cancer, Metabolic/Obesity and Cardiovascular)

There are some factors in lifestyle due to developing urban life which have a tight and direct link to health. The most important factor is diet. Using fast foods and junk foods, causes nutrition problems like obesity and cardiovascular disease (23). Addressing the issue of lifestyle practices from both a material and a spiritual perspective is absolutely essential.

Given the growth in chronic diseases in the recent decade in developing countries which has led to increased health problems, such as obesity (5), cardiovascular diseases and diabetes, modifying various aspects of lifestyle in all the classes of the society are necessary. Cardiomyocyte metabolism is under circadian control (20), and circadian and diurnal rhythms are, in turn, observed in blood pressure, heart rate, and platelet aggregation, as well as in the incidence of multiple categories of CVD (24). This has led to investigation of the link between circadian disturbance and cardiovascular risk factors and health outcomes. Shift work is associated with both circadian disruption and sleep loss. Common estimates of under 20% have been reported for shift work in the industrial world (25).

About 25% of the world population suffers from metabolic syndrome. These patients who have a five-fold higher risk of developing type 2 diabetes are exposed to heart attacks or strokes by at least a factor of three (26). According to WHO, 422 million adults are estimated to have type 2 diabetes worldwide and it is predicted that diabetes deaths will double by 2030 (27).

Research has not shown any disturbance of circadian rhythm due to unregulated lifestyle will increase the incidence rate of some diseases such as different kinds of cancers and metabolic disorders. “Efforts are underway to develop approaches in chronobiology and pharmacology to modify the period, phase or amplitude of circadian clocks to improve human health” (28).

### Circadian Rhythm and Psychological/Behavioral Disorder

**Fig. 5:**
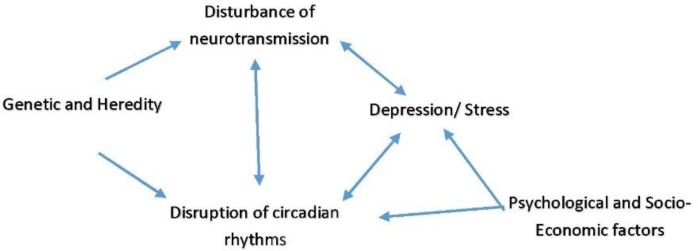
Potential model of depression. The reciprocal relationship between the disruption of circadian rhythms and behavioral disorders may be either cause or effect of a depressed affective state, and it is influenced by genetic, neurochemical, and neuroendocrine factors (29)

Depression as a mental illness which leads to medical issues and health disorders have a series of negative effects on individual’s functions including feeling and thinking. It is originated from genetic, hormonal or physiological factors, or it may be made by stressful environments or life conditions (29) (Fig. 5). Moreover, the disruption of circadian rhythms can contribute to the beginning of depression (16). Though the etiology of depression is complicated, circadian factors might have a significant effects in the process. As a disturbance of circadian rhythm could be due to lighting conditions and lifestyle in individuals who were exposed to a wide range of mood disorders including impulsivity, mania and depression (30). “As risk of violent crime is increased in individuals with depression after adjustment for familial, sociodemographic and individual factors in two longitudinal studies” (31), its results could negatively affect public health through physical impairments, mental/behavioral/job impairments/educational and risky behaviors (32).

### Geographic Events and Circadian Rhythm

Some other important factors which affect the circadian rhythms are geological cycles like the length of daylight and darkness in different seasons which originate from earth’s movements consist of rotation and transition. These geographic events influence on adaptation processes to all organisms where they have to adjust their physiology to the environmental changes (16). In winter that day becomes shorter and night gets longer than spring and summer, depression is increased due to a diminished sunlight exposure. This transient mood disorder has been called seasonal affective disorder (SAD). People who live in the countries where seasonal changes are more significant, that is, days are shorter and sunlight is diminished during the day in the winter, are extensively exposed to depression (16).

### Cosmic Factors and Circadian Rhythm

**Fig. 6:**
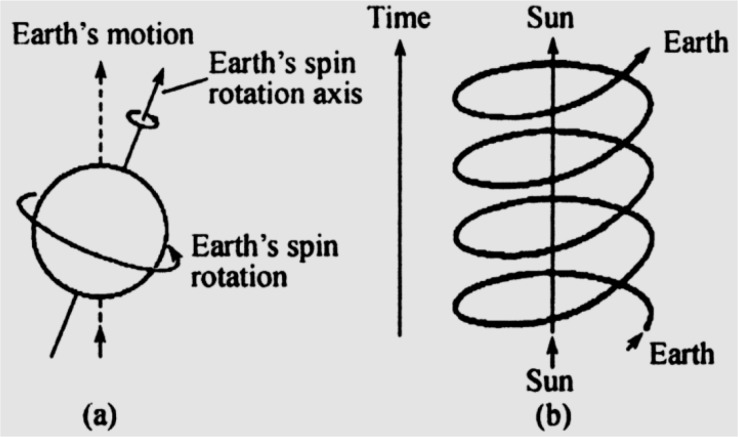
The natural right-handed helical Earth’s orbital chirality and its force field. It is produced by both the Earth’s right-handed helical rotation around its spin rotation axis (a) and the Earth’s helical revolution around the Sun (b) (38)

In the solar system, the combination of the Earth’s spinning and revolution (axial vector) and the Earth’s motion toward Vega (polar vector) forms the right-handed superhelical motion with circadian, seasonal and annual periods in space-time. It creates the Earth’s orbital chirality (EOC) and its corresponding EOC force field in which terrestrial life exist (33, 35) (Fig. 6). In terms of force field of EOC is chiral and right-handed helical, with seasonal and annual rhythms, stability of the right-handed helical secondary structures in biological molecules are more than the left-handed helical ones (36).

The chiral interactions of the rhythmic EOC force field with the chiral biomolecules could give rise to the periodicity of the biomolecular stability to result in the origin of biological rhythms in terrestrial living systems (36). It was also demonstrated that the EOC force field could cause the rhythms and symmetry breaking in radioactive processes (37). It indicates the EOC effects should be universal on different levels (38).

A model presented by Bilhamand and Bendick, predicted a higher activities in large earthquake in 2018 originate from a very slowly changes in the rate of the Earth’s spins. “Although we never notice it, the Earth’s spin does change ever so slightly” (39). Regarding this, EOC force field would be changed which affects biological rhythms in organisms.

In addition to the above mentioned factors, there are other factors such as high altitude and changing ecosystem which definitely affect biological rhythms of all animals especially human. Although it has not reported certain experimental and scientific documents to prove them yet, the given impression of living organisms’ lives from them do seem inevitable.

## Conclusion

There are a variety of factors consisting of lifestyle, geological events and biological molecules which impact on circadian rhythms. As there is a direct relationship between uncertain sleep-waking times and disrupting circadian rhythm, having unregulated schedules can increase incidence risk of chronic diseases. On the other hand, in developing countries, people do not pay attention to determine their daily schedules somehow and incidence risk of chronic diseases is more prevalent. So, the most important factor is lifestyle which effects other factors. Hence, we have to focus on changing our lifestyle and life policies in a positive and healthy ways. Although, lifestyle is based on personal choices and identities, once publicized, it cannot be analyzed out of its social and cultural context. At the micro level, the personality and biological and psychological characteristics of the individual, family, friends, school, and society affect the individual’s daily life and lifestyle. At the macro level, the city and the larger world the individual inhabits, the media, the highly active social and political atmosphere in which the individual is involved and the cultural climate of the society in which he or she lives affect lifestyle and its changes. Politicians and governments should thus not be indifferent to lifestyle changes and should encourage researchers to conduct studies on the subject. Also, all terrestrial living beings, even us, are living in the cosmic natural events that it is impossible for humans to adapt to their affects within a short term, and they will disturb human’s circadian rhythms and cause physiological problems.

## Ethical considerations

Ethical issues (Including plagiarism, informed consent, misconduct, data fabrication and/or falsification, double publication and/or submission, redundancy, etc.) have been completely observed by the authors.
